# Investigation of the Neutralizing Behaviors of Cement-Based Materials Using a New pH Indicator Formulated from February Orchid Petals

**DOI:** 10.3390/ma15228033

**Published:** 2022-11-14

**Authors:** Dong Cui, Xiaohan Shi, Wenya Liu, Keren Zheng, Guangji Yin, Jing Wang, Guantong Han, Yi Wan, Junsong Wang, Wenting Li

**Affiliations:** 1 School of Science, Nanjing University of Science and Technology, Nanjing 210094, China; 2 School of Environmental and Biological Engineering, Nanjing University of Science and Technology, Nanjing 210094, China; 3 Department of Civil Engineering, Central South University, Changsha 410075, China; 4 School of Civil and Transportation Engineering, Ningbo University of Technology, Ningbo 315211, China; 5 Key Laboratory of Advanced Civil Engineering Materials, Ministry of Education, Tongji University, Shanghai 201804, China

**Keywords:** carbonation, leaching, sulfate attack, cement-based materials, pH indicator, neutralization, February orchid

## Abstract

Investigation of the neutralizing behavior of concrete is essential, as it can help reveal the durability properties of concrete structures. In this paper, anthocyanin extracted from February orchid (F. orchid) petals was used to characterize the neutralized (carbonated, leached, and sulfate-attacked) regions of cement-based materials. The durability of F. orchid indicator was evaluated through comparison between discoloring behaviors of fresh and aged F. orchid indicators, and the capability of the new indicator in neutralization characterization was then verified by combining indicator (phenolphthalein, malachite green, indigo carmine, or thymolphthalein) spray, X-ray computed tomography (CT), and the X-ray attenuation method (XRAM). The result in the present study showed that, with a lower color intensity as compared to phenolphthalein/thymolphthalein, F. orchid indicator was less preferable in studying carbonation but a better choice in characterizing leaching and sulfate attack of cement-based materials. In addition, a sharp carbonation front was revealed in the present study, suggesting that the carbonation process in this study was controlled mainly by diffusion. For leaching and sulfate attack, the broader fronts revealed suggested that both processes were co-controlled by diffusion and reaction. The current work serves as a ‘leap’ toward the application of natural pigments in analyzing the durability of concrete structures.

## 1. Introduction

Structural concretes used in Chinese national projects (e.g., Tibet-entering project and sea-crossing project) are facing dual challenges of harsh serving environments and severe loading conditions [1,2,3]. Therefore, there is an urgent need to cast concretes with high strength, satisfying resilience, and long durability [4,5]. Unfortunately, serving as a typical brittle material, the cracking of concrete in-site is almost inevitable [6,7]. Subsequently, corrosive media, such as CO_2_, water, and sulfate ions, enter concrete through these cracks, causing degradation and ultimately failure of the concrete structure ahead of the designed service life [3,8].

During the invasion of corrosive media, the alkaline substance formed during hydration is consumed, which significantly reduces the pH of the pore solution in concretes [9,10]. Therefore, pH reduction (neutralization) has been universally adopted as a calibrator, reflecting the deterioration degree of concrete structures [11,12]. In past decades, phenolphthalein was the most-used pH indicator for these neutralization analysis, based on which the durability performance of concrete subjected to both carbonation [13] and leaching [14] was evaluated. However, the deficiency of phenolphthalein was quite clear: first, phenolphthalein is of potential carcinogenicity [15], so it is not entirely safe to handle the indicator; secondly, a sufficient reduction in pH is the prerequisite to trigger the discoloration of phenolphthalein, but the discoloration pH of phenolphthalein may not be completely the same as that of the neutralization front [11]. As a consequence, phenolphthalein may underestimate the neutralized area [16,17]. The deficiency of phenolphthalein was especially significant when investigating sulfate-attacked concrete, where the indicator was almost ‘blind’ due to the limited reduction in inner pH within eroded concretes [18].

To get its house in order, attempts to ‘dig out’ more suitable pH indicators for durability analysis never stopped. In past decades, pH indicators, such as thymolphthalein [19,20,21,22,23,24,25], tropaeolin O [25,26], Nile blue [27], alizarin yellow R [25,28,29,30,31,32], and indigo carmine [32], have all been successfully adopted for the investigation of carbonation in cement-based materials. Due to a slightly higher discoloration pH as compared to phenolphthalein, thymolphthalein [19], tropaeolin O [26], indigo carmine [32], and alizarin yellow R [29,31,33] all presented larger but still reliable carbonation results. Considering that the partial carbonated zone (favors the corrosion of rebar [34]) commonly existed in reinforced concrete but was mostly overlooked using phenolphthalein [35], the adoption of the above-mentioned pH indicators would effectively mitigate the deficiency, and a more accurate durability analysis could then be made. Better yet, previous research validated that, when handling geopolymer concretes, the carbonation boundary read from alizarin yellow R would be clearer than that read from phenolphthalein [29]. In addition, through mixing of different chemicals (e.g., combination of cresol red and thymol blue [36], or rainbow indicator [22]), the discoloration pH of the formulated pH indicator could be precisely designed, and the phenomenon of difficult observation (e.g., early carbonation) could also be clearly captured [25].

Despite their effectiveness and lower toxicity, the above-mentioned pH indicators are still not completely harmless. For instance, pH indicators such as thymolphthalein, alizarin yellow, and indigo carmine may irritate human skin or eyes if handled incorrectly. Therefore, to further enhance the safety of durability analysis, the development of a new pH indicator is still necessary. Recently, inspired by biological work, natural pigments such as curcumin [37] and anthocyanin [34,35] were also successfully adopted for the durability analysis. Compared with former pH indicators, anthocyanin is completely toxicity-free (edible) and is, thus, applicable, especially in circumstances with rigid safety requirements. Regardless, previous work has concentrated merely on the introduction of these natural pigment-based pH indicators, but a limited concentration has been given on their reproducibility, reliability, and durability. In addition, most former work focused only on carbonation, while for other important neutralization processes, such as leaching or sulfate attack, the capability of the indicator spray method in their characterization still remained unknown.

In this paper, a new pH indicator based on anthocyanin extracted via February orchid (F. orchid, also known as ‘*Orychophragmus violaceus*’ [38]) petals was formulated as the pH indicator. The durability of the new F. orchid indicator was evaluated, and its capability in tracing carbonation, leaching, and sulfate attack was all systematically investigated. In general, pigment in F. orchid petals (mainly anthocyanin) is very sensitive to the variation in environmental pH [39]; thus, it is suitable as a pH indicator. In addition, serving as the most-common ground cover plant in Asia, the budget of F. orchid indicator can be largely reduced compared with anthocyanin sourced from crops, vegetables, or fruits [35]. Moreover, the F. orchid plant contains no toxicity to the human body, so it is safer to use [40]. Through a combination of phenolphthalein/malachite green/indigo carmine/thymolphthalein spray, computed tomography (CT) scan, and the X-ray attenuation method (XRAM), the reliability of the new F. orchid indicator in characterizing neutralization (carbonation, leaching, and sulfate attack) was verified. The current work serves as a ‘leap’ toward the application of natural pigment in the durability design for concrete structures.

## 2. Materials and Methods

### 2.1. Formulation of Anthocyanin Indicator

**Figure 1:**
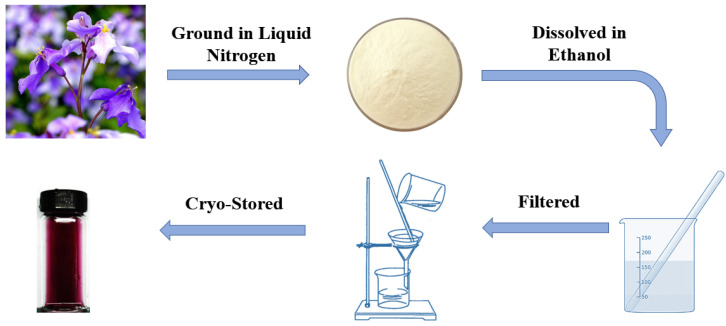
Formulating procedure of F. orchid indicator.

The detailed formulating procedures are as follows. Fresh F. orchid flowers from the university campus were collected. The petals were then separated and refrigerated at 4 °C (most-widely adopted temperature for the preservation of organic compounds [41,42]). Next, petals were rinsed with de-ionized water, mixed with liquid nitrogen in a mortar, and ground into powder with a pestle. Subsequently, 50 g of frozen powder was taken and dissolved in 100 mL of ethanol. The mix was settled for no less than 24 h to guarantee full dissolution. Finally, the ethanol solution was filtered and condensed under reduced pressure to complete the formulation process (see Figure 1). The F. orchid indicator was stored in a sealed dark glass bottle. Note that both fresh and aged (cryopreserved at approximately −5 °C for 3 months) pH indicators were prepared in the present study, which promises examination of the indicator’s durability.

### 2.2. Sample Preparation and Neutralization Test

Paste samples were cast using P⋅II cement manufactured by Xiaoyetian cement factory. The water-to-cement ratio was 0.45. Molds with an inner diameter of 4 cm were used, and paste specimens after being cast were covered with a plastic sheet, left ambiently for one day. Next, all de-molded pastes were standard-cured (temperature: 20 ± 1 °C, relative humidity: ≥95%) for another month. After curing, specimens were sent for accelerated carbonation, leaching, and sulfate attack. The detailed procedure for each test is as follows:

Carbonation: Pastes were cut into 5 mm slices. Next, the slices were mass-balanced under a humidity of about 60% for 3 months, and nitrogen was injected periodically to avoid air carbonation. After that, the top and bottom faces of each slice were covered by epoxy resin, leaving the side face for accelerated carbonation. The test was performed following Ref. [10]: in a chamber with constant climate (humidity: 60 ± 5%, temperature: 20 ± 2 °C, and CO_2_ concentration: 3 ± 1%).

During carbonation, calcium-bearing materials (mainly Ca(OH)_2_) were consumed, incurring a reduction in inner pH, as shown in Equation (1) [9].

(1) $$\mathrm{Ca}{\left(\mathrm{OH}\right)}_{2}+{\mathrm{CO}}_{2}\to {\mathrm{CaCO}}_{3}\left(↓\right)+H_{2}O$$

Leaching: This test was performed following Ref. [14]. Similar to carbonation, the top and bottom faces of each slice were sealed with epoxy resin. All slices were immersed in 6 M NH_4_Cl solution, and the pH of the leaching solution was examined periodically to achieve constancy. The temperature of the leaching solution was maintained at 20 ± 2 °C.

During accelerated leaching, the reaction between Ca(OH)_2_ and NH_4_Cl was promoted, forming highly soluble CaCl_2_ and gaseous NH_3_, as shown in Equation (2) [14].

(2) $$\mathrm{Ca}{\left(\mathrm{OH}\right)}_{2}+2{\mathrm{NH}}_{4}\mathrm{Cl}={\mathrm{CaCl}}_{2}+2{\mathrm{NH}}_{3}\left(↑\right)+2H_{2}O$$

Sulfate attack: Ref. [12] was used as the reference. The top and bottom faces of pastes were sealed with epoxy resin. Each slice was then conditioned in a vacuum oven of 45 °C for 14 days. Next, samples were immersed in 3.0% by mass of sodium sulfate (Na_2_SO_4_) solution. The temperature of the sulfate attack solution was fixed at 20 ± 2 °C.

The deterioration mechanism under sulfate attack was complex. Primarily, sulfate reacted with calcium-bearing materials (mainly Ca(OH)_2_), forming calcium sulfate (CaSO_4_). Next, CaSO_4_ continued to react with solid calcium aluminate hydrate (CAH), forming ettringite (3CaO⋅Al_2_O_3_⋅3CaSO_4_). The main processes are depicted in Equations (3) and (4) [12].

(3) $${\mathrm{Na}}_{2}{\mathrm{SO}}_{4}\cdot 10H_{2}O+\mathrm{Ca}{\left(\mathrm{OH}\right)}_{2}\to {\mathrm{CaSO}}_{4}\cdot 2H_{2}O+2\mathrm{NaOH}+8H_{2}O$$

(4) $$3\left({\mathrm{CaSO}}_{4}\cdot 2H_{2}O\right)+4\mathrm{CaO}\cdot {\mathrm{Al}}_{2}O_{3}\cdot 12H_{2}O+14H_{2}O\to 3\mathrm{CaO}\cdot {\mathrm{Al}}_{2}O_{3}\cdot 3{\mathrm{CaSO}}_{4}+\mathrm{Ca}{\left(\mathrm{OH}\right)}_{2}$$

### 2.3. Testing Methods

#### 2.3.1. High-Performance Liquid Chromatograph (HPLC)

A Shimadzu LC-20AT high-performance liquid chromatograph (HPLC) equipped with a PDA detector was used in this study. F. orchid indicator was incubated at 30 °C in an InertSustain C_18_ column (4.6 × 250 mm, 5 μm), with a flow rate of 1 mL/min. C in C_18_ stands for carbon atom. C_18_ is the abbreviation of the octadecylsilyl bonding phase. It is easy for hydrophobic samples to interact with C_18_ functional groups in the chromatographic process to generate chromatographic retention, and the retention strength is positively related to its hydrophobicity. The detection wavelength was 340 nm. 0.1% formic acid water (solvent A) and acetonitrile (solvent B) were used as the gradient elution, starting from 5% solvent B and linearly increasing to 100% solvent B within 20 min. Next, the elution was maintained at 100% solvent B for 10 min and then re-equilibrated with 5% solvent B for another 10 min.

#### 2.3.2. Indicator Spray Test

The spray test was performed to trace neutralization, and phenolphthalein, malachite green, indigo carmine, thymolphthalein, and F. orchid indicator were used separately in the present study. The cross-section of the partly neutralized specimen was exposed first (carbonated specimen: split; leached or sulfate-attacked specimen: cut through a diamond saw). The newly exposed cross-section of the carbonated specimen was sprayed immediately with F. orchid indicator or phenolphthalein, while for the leached or sulfate-attacked specimen, the cross-section was flushed first with de-ionized water for 2 days (removal of remaining leaching/sulfate-attacking agents), and then sprayed with F. orchid indicator or phenolphthalein to trigger discoloration.

#### 2.3.3. X-ray Computed Tomography (CT)

X-ray computed tomography (CT) was used to characterize the neutralized areas. The CT used here was manufactured from YXLON Company in Germany. The scanning parameters were set still during multiple scans (working voltage: 195 kV; working current: 0.3 mA; effective resolution: 60 μm; filter quality: 0.5 mm aluminum sheet). To improve the signal-to-noise ratio, an average of 6 projections (300 ms per projection) were recorded during CT reconstruction.

#### 2.3.4. X-ray Attenuation Method (XRAM)

**Figure 2:**
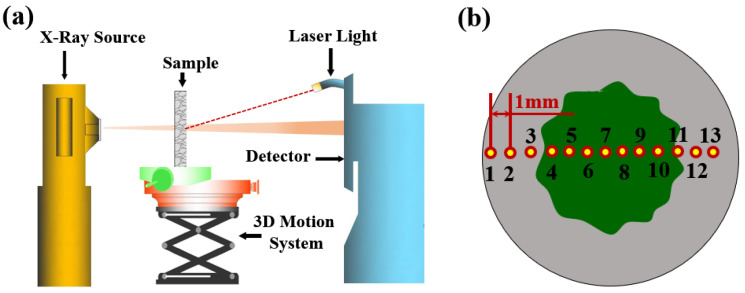
XRAM apparatus and sampling position. (**a**) Schematic of XRAM apparatus; (**b**) sampling location.

The X-ray scanner used in this study was produced by Aoshi Electronic Technology Co., Ltd. in Dongguan City, Guangdong Province, China. The scanning parameters were set still during multiple scans (working voltage: 70 kV; working current: 0.6 mA; filter quality: 0.5 mm aluminum sheet). Laser light was used to geometrically align the position of the specimen from multiple scans, and a 3D moving table was adopted to control the motion of the tested sample (see Figure 2a). For each neutralized sample, 13 points horizontally from left to right, passing through the center of the scanned sample, were selected as the testing points. The horizontal distance between adjacent points was 1 mm, and the distance from point 1 to the left corner of the tested specimen was set as 1 mm (see Figure 2b).

The detailed procedures for porosity measurement are as follows. First, put the sample in a container holding cooled water (boiled first to reduce the amount of dissolved gas), and then connect the container to one 2.0 CFM vacuum pump. Vacuum the container through the pump continuously for two weeks, until the sample mass no longer changes. Next, measure the attenuation coefficients of the saturated sample at the sampling locations (see Figure 2b), and put the sample afterward in a vacuum oven of 45 °C for another two weeks. Subsequently, measure the attenuation coefficients of the dried sample from the same sampling locations. The local porosity of the tested sample was obtained through the altered attenuation coefficient during a dual scan, as shown in Equation (5).

(5) $$P=\frac{A_{sat}-A_{dry}}{l\mu_{\omega }\rho_{\omega }}\times 100\%$$

where $A_{sat}$ and $A_{dry}$ are the attenuation coefficients of an identical region in the saturated and dried state, respectively; *l* is the thickness of the scanned sample; $\mu_{\omega }$ and $\rho_{\omega }$ are the mass attenuation coefficient and mass density of water, respectively.

## 3. Results

### 3.1. Discoloring Behavior of F. Orchid Indicator

**Figure 3:**
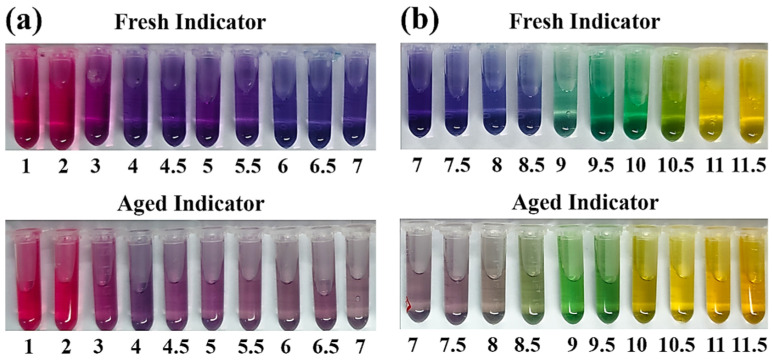
Discoloring behavior of fresh and aged F. orchid indicators exposed to pH ranges of (**a**) 1.0 to 7.0 and (**b**) 7.0 to 11.5.

Figure 3 shows the discoloring behavior of F. orchid indicator in the pH range of 1.0 to 11.5. Obviously, the indicator presented a wide range of colors when facing environments of varying pH: when the surrounding pH was lower than 4, the indicator presented as pink. The indicator color at the acidic state was close to that of F. orchid petals, which implied that the pH of the cell solution for F. orchid petals was acidic, and the acidic condition was, therefore, expected to be beneficial for the durability of F. orchid indicator. When the surrounding pH increased to a range of 4.0 to 8.5, due to the reversible formation of quinonoidal anhydrobase [43,44], the color of fresh indicator shifted to blue, while for aged indicator, a similar discoloration with weaker color intensity was presented, signifying partial degradation of the indicator. When the surrounding pH was further enhanced to a level higher than 9, the color of green started to emerge. Noticing that the discoloration pH was fixed at 9 for both fresh and aged F. orchid indicators, the reliability of F. orchid indicator, even partly degraded, was validated as suitable for neutralization characterization. Eventually, when facing an alkaline environment with a pH higher than 10, F. orchid indicator changed to yellow, demonstrating the irreversible formation of chalcone (degradation) [45].

According to the discoloration behavior of aged indicator, the stability of F. orchid indicator was confirmed as less effective compared to phenolphthalein. Despite that, recent studies [34] have proven that anthocyanin indicator would be more durable if formulated immediately after plucking and stored constantly under lower temperature. Nonetheless, as the discoloration pH of F. orchid indicator, even partly degraded, was fixed in the pH range of 7 to 10, F. orchid indicator was verified as applicable for pH analysis.

**Figure 4:**
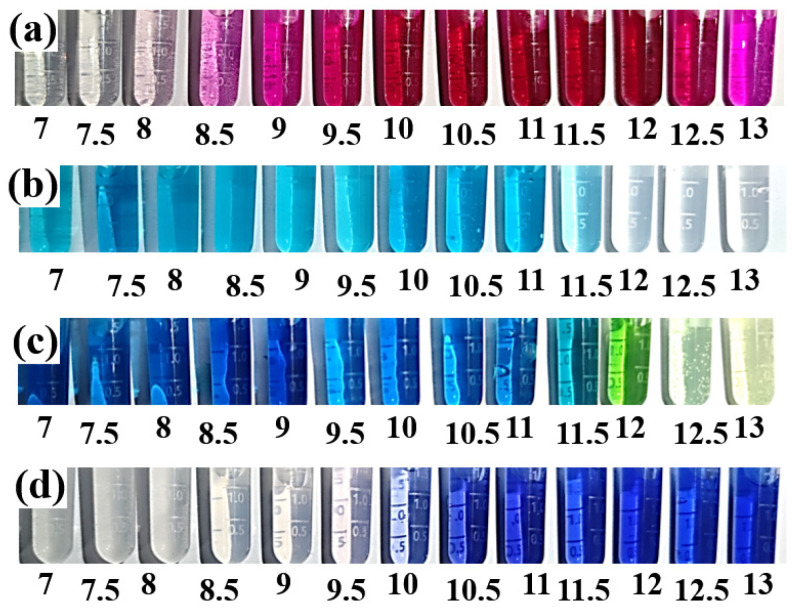
Discoloration behavior of (**a**) phenolphthalein, (**b**) malachite green, (**c**) indigo carmine, and (**d**) thymolphthalein exposed to varying pH.

To better evaluate the discoloration behavior of F. orchid indicator, similar discoloration tests were performed on four pH indicators used worldwide in durability analysis (phenolphthalein, malachite green, indigo carmine, and thymolphthalein), as shown in Figure 4. From the pH of 7.0 up to 13, phenolphthalein, malachite green, indigo carmine, and thymolphthalein turned from colorless, light blue, dark blue, and colorless, respectively, to pink, colorless, yellow, and blue. The discoloration pHs of phenolphthalein, malachite green, indigo carmine, and thymolphthalein in the pH range of 7 to 13 were approximately 8.5, 12, 12, and 10, respectively. Apparently, the discoloration pH of anthocyanin (about 9) was in between those of phenolphthalein (about 8.5) and thymolphthalein (about 10), but significantly lower those of malachite green (about 12) and indigo carmine (about 12). Note here that the pH of the carbonation front is within the pH range of 8.5 to 10 [34], and indigo carmine and malachite green can present larger but sill reliable carbonated areas due to their higher discoloration pH [19,26]. For leaching and sulfate attack, the use of pH indicators was less reported, and the effectiveness of pH indicator, therefore, needed further experimental validation.

### 3.2. Chromatograms Obtained from HPLC

**Figure 5:**
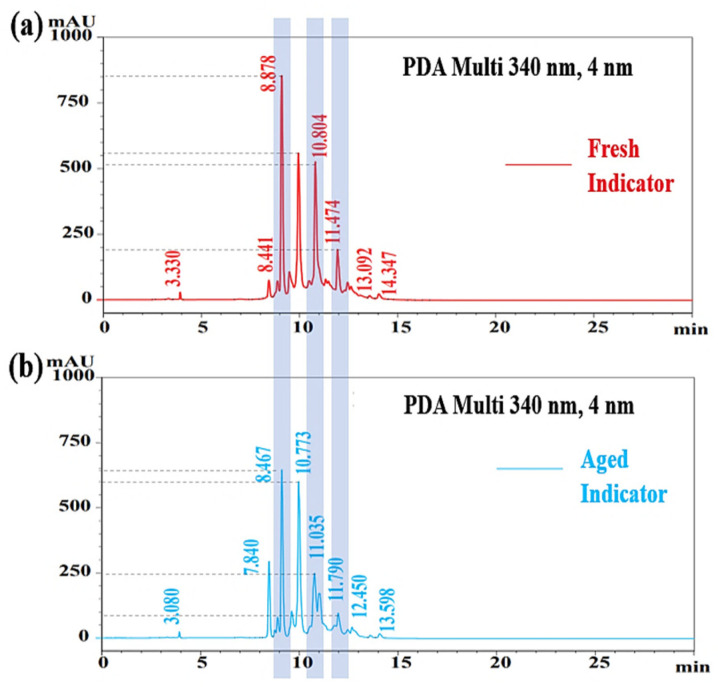
Chromatograms of (**a**) fresh and (**b**) aged F. orchid indicators.

Figure 5 shows the chromatograms of fresh and aged F. orchid indicators. The chromatograms were obtained from high-performance liquid chromatography (HPLC), which was essential in present study. This was because HPLC could help balance the concentration of the new developed indicator, which was achieved through holding the height of the 10 min peak at 550 mAU. As shown in Figure 4, even after three months of cryopreservation, the height of the 10 min peak remained almost unchanged, which verified that the adopted peak was suitable as the concentration calibrator. In addition, chromatograms obtained from HPLC could also be used to evaluate the stability of F. orchid indicator. Although each peak in both Figure 5a,b was related to each other, the peak heights of anthocyanins (marked by gray bars) were significantly reduced after three months of cryopreservation. The result evidenced the degradation of F. orchid indicator, which complied with the discoloring behavior in Figure 3.

### 3.3. Neutralized Regions Characterized by Varying Indicators

**Figure 6:**
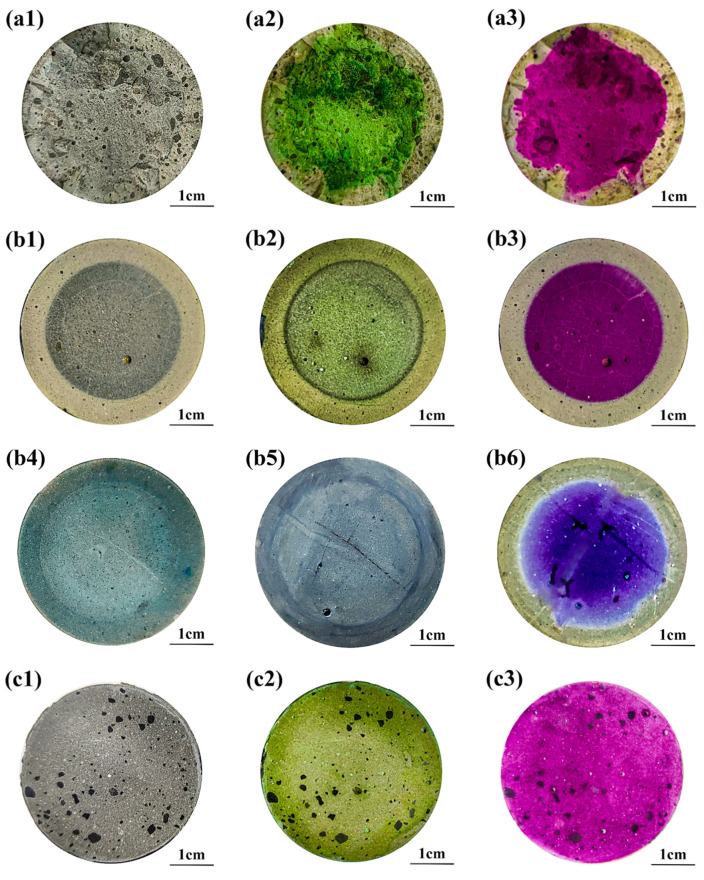

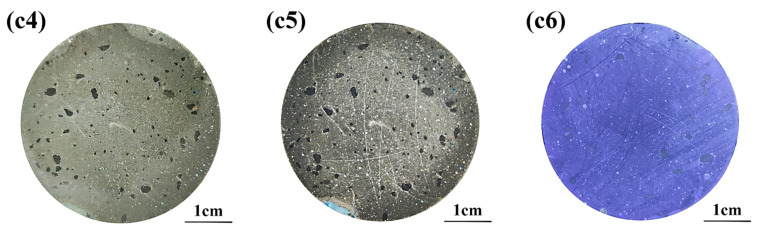
Partly neutralized sample before and after pH indicator spray. ‘**a**’, ‘**b**’, and ‘**c**’ stand for partly carbonated, leached, and sulfate-attacked sample, respectively; suffix ‘**1**’, ‘**2**’, ‘**3**’, ‘**4**’, ‘**5**’, and ‘**6**’ stand for specimens before spraying and spraying with anthocyanin, phenolphthalein, malachite green, indigo carmine, and thymolphthalein, respectively.

Cross-sections of partly carbonated, leached, and sulfate-attacked paste specimens were pretreated (see Section 2.2) and sprayed with fresh F. orchid indicator, phenolphthalein, malachite green, indigo carmine, and thymolphthalein. The initial cross-sections of neutralized specimens before spraying are given in Figure 6(a1–c1). Apparently, the carbonated and sulfate-attacked areas were overall invisible without spraying, but the partly leached area could be discerned already. The discernable leaching front without spraying was reasonable, as calcium-bearing materials were drastically lost during leaching, and the microstructure of the leached area, therefore, was significantly coarsened [46,47]. For the partly carbonated or sulfate-attacked specimen, the change in microstructure was less significant, so the difference between the carbonated/sulfate-attacked and non-carbonated/non-sulfate-attacked area was less recognizable [48].

The cross-sections of the partly carbonated specimen after spraying with F. orchid indicator and phenolphthalein are shown, respectively, in Figure 6(a2,a3). After spraying with F. orchid indicator, the carbonated (boundary) and non-carbonated (core) region turned khaki and green, respectively, while after spraying with phenolphthalein, the non-carbonated region changed into pink/fuchsia, and the carbonated region remained colorless. As the pH of pore solution gradually reduced from higher than 12 to lower than 9 during carbonation [10], the reduction in pH was sufficient to incur discoloration of both F. orchid indicator (see Figure 3) and phenolphthalein. For indigo carmine and thymolphthalein, their effectiveness in carbonation characterization has been confirmed previously [19,20,21,22,23,24,25,32]. Nonetheless, note that the carbonation front shape was irregular after carbonation, and an accurate recognition of the carbonated area appears more difficult, while essential. Therefore, when dealing with carbonation, phenolphthalein/thymolphthalein appear more suitable over F. orchid indicator, as they illustrate a clearer boundary area due to their high color intensities. For the new F. orchid indicator, even though the indicator is also workable, the carbonated area read will be less clear compared to phenolphthalein or thymolphthalein.

With the continuous loss of calcium-bearing materials, a severe reduction in inner pH occurred, and similar discoloring behaviors were observed after spraying with anthocyanin, phenolphthalein, and thymolphthalein in leaching tests (see Figure 6(b2,b3,b6)). For the cross-section sprayed with malachite green or indigo carmine, the leached area after spraying appeared even vaguer than that before spraying. The result was reasonable considering that the discoloration pH of malachite green or indigo carmine was about 12 (see Figure 4), and the leached and non-leached area should appear blue and colorless (yellow), respectively, when spraying with malachite (indigo carmine). Obviously, the leached area after spraying (tinged with blue) reduced the color difference between leached and non-leached areas, making the process less recognizable. Compared with the carbonation front, the shape of the leaching front was more regular, and this discrepancy between shapes of carbonation and the leaching front was also reported in the literature [47,49]. The varying serving conditions are referred here to explain the difference: the leaching test was performed underwater, while carbonation was mostly carried out under an intermediate humidity (usually of 40–60% [49,50]). As pores in cement-based materials remained fully saturated throughout leaching, drying shrinkage caused by humidity evolution was largely avoided, and a regular-shaped leaching front was, thus, generated due to the absence of cracking. For carbonation, because the pores were not fully saturated, the fluctuations in inner humidity, drying shrinkage [51,52], and carbonation shrinkage [53] were inevitable, both of which incurred cracking. Later, CO_2_ was preferable to diffuse through the cracks, forming an irregular-shaped carbonation front.

For sulfate attack, it is interesting to point out that only F. orchid indicator and indigo carmine appeared effective, while none of the remaining pH indicators worked. A possible reason is given here: even though the inner pH of the sulfate-attacked area decreased, the reduction was insufficient to trigger discoloration of any pH indicators presented in Figure 6 [54,55]. Therefore, similar to the non-eroded area, the sulfate-attacked area displayed a**s** green, pink, colorless, yellow, and blue, respectively, after spraying with F. orchid indicator, phenolphthalein, malachite green, indigo carmine, and thymolphthalein. Regardless, sulfate attack deteriorated the microstructure of cement-based materials [54], and pigment could then infiltrate into the sulfate-attacked area, causing color accumulation (appearance of deep green and deep gray, respectively, for F. orchid indicator and indigo carmine). For phenolphthalein or thymolphthalein, even though pigment infiltration was also performed, the color accumulation was less observable considering the high color intensity of the indicator itself. As for malachite green, the pigment accumulation was also non-observable, as the indicator appeared colorless within the sulfate-attacked area. Color accumulation can also explain the dark ring connecting the non-leached and leached area (see Figure 6(b2)), as the ring zone also acquired insufficient pH reduction and a porous microstructure, and infiltration of pigment within the leaching front was equally possible. The assumption here is further verified by the microstructural result in the following section.

Compared between Figure 3 and Figure 6, the discoloring behavior in both figures was different. The inner pH of the non-neutralized region was higher than 12 [46,50]; thus, it should exhibit a yellow color after spraying with F. orchid indicator according to Figure 3. However, in Figure 6, green instead of yellow was presented. The discrepancy illustrated a postponed emergence of indicator expiration, which was most probably caused by the varying ratio of the F. orchid indicator volume to the examined solution volume: unlike phenolphthalein, anthocyanin was highly unstable and, thus, inclined to degrade (turning yellow) under a high-alkaline environment. In Figure 3, only a few drops of F. orchid indicator were dropped into the tested solution, so anthocyanin with complete contact to the test solution lost its efficiency immediately, while in Figure 6, only the exposed cross-section was in contact with the F. orchid indicator, so anthocyanin under that condition could survive longer, hence presenting as green. In addition, after spraying with F. orchid indicator, it was also noticed that the color intensity of the non-leached/non-sulfate-attacked region was weaker compared to that of the non-carbonated region, even though the pH within three regions should be similar. The incompatibility was most likely caused by higher humidity levels in specimens exposed to leaching/sulfate attack: as leaching and sulfate attack tests were conducted underwater, higher amounts of water remained in the specimen. After spraying, the F. orchid indicator could be diluted by water in pore solution, leading to a weaker color intensity in the partly leached/sulfate attacked specimen.

### 3.4. Validation of F. Orchid Indicator’s Reliability

**Figure 7:**
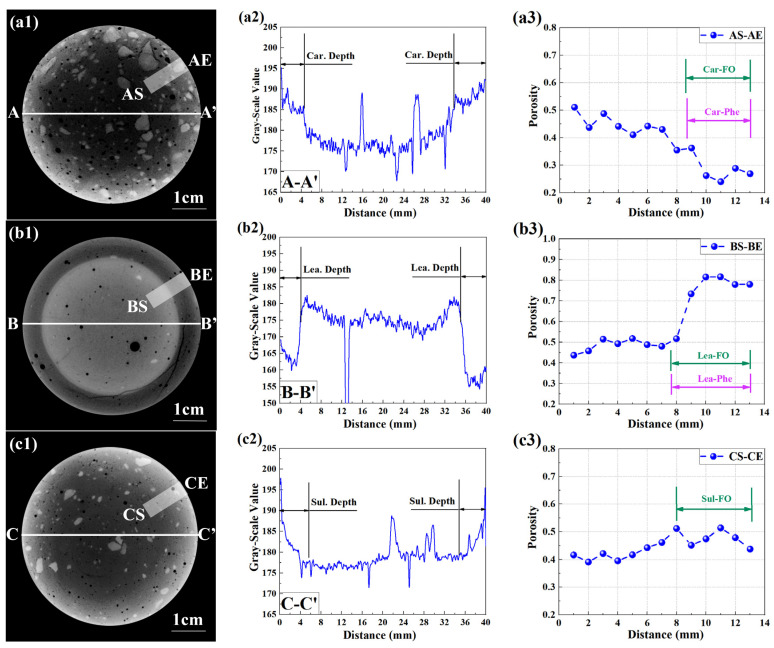
Neutralized regions based on CT and XRAM. ‘**a**’, ‘**b**’, and ‘**c**’ stand for partly carbonated, leached, and sulfate-attacked sample, respectively; suffix ‘**1**’ stands for the rendered cross-section based on CT; suffix ‘**2**’ and ‘**3**’ stand, respectively, for the 1D grayscale value and 1D porosity distribution. FO: F. orchid indicator; Phe: phenolphthalein.

Partly neutralized specimens were further scanned with CT, based on which the reliability of F. orchid indicator was verified. Figure 7(a1–c1) present the typical cross-sections rendered from the partly carbonated, leached, and sulfate-attacked specimen, respectively. Note that each image in Figure 7 was aligned with the related image in Figure 6, and both images can be drawn directly for comparison.

The attenuation coefficient of cement-based material increased after carbonation or sulfate attack. On the contrary, compared with the non-leached core, the leached surface was significantly darker, highlighting the drastic loss of calcium-bearing materials. Despite the difference, carbonated, leached, and sulfate-attacked regions were all clearly revealed through CT scans, and the result showed high consistency with that revealed through spraying with F. orchid indicator, which served as compelling evidence that the newly developed pH indicator was trustable in neutralization characterization.

To quantitatively evaluate the reliability of F. orchid indicator, linear profiles of grayscale values collected from 2D renderings were plotted (see Figure 7(a2–c2)). The grayscale values of non-carbonated, non-leached, and non-sulfate-attacked regions were in the range of 170–180, and randomly encountered peaks and valleys were observable on 1D profiles, representing the unhydrated cement particles or macro-pores. The grayscale values of the carbonated, leached, and sulfate-attacked region were in the range of 185–190, 155–170, and 175–190, respectively, which quantitatively revealed an increased attenuation coefficient by CO_2_ or sulfate binding, but a reduced one by leaching. In addition, grayscale values at both ends of the 1D profile were larger than that of the inner neutralized area, which was due to the artifact of beam hardening (inevitable during CT scan [56]). Nonetheless, a sharp increase (reduction) was observed on the 1D carbonation (leaching) profile, which suggested that the carbonation (leaching) in the present study was a diffusion-controlling progress. Based on a similar carbonation (leaching) depth read from the 1D grayscale value profile and from local pH, the reliability of F. orchid indicator in the neutralization characterization was confirmed again. An exception was encountered in sulfate attack, where no clear front was observed on its 1D profile. The result highlighted the fact that sulfate attack in the present study was not solely controlled by diffusion. The sulfate-attacked depth could still be read as approximately 5 mm, which agreed with that based on spraying with F. orchid indicator.

The X-ray attenuation method (XRAM) was adopted for further validation from a microstructure perspective. The method has been successfully applied by the same authors for the investigation of carbonation [51,53] and leaching [57], where the local porosity was obtained through a dual scan of an identical specimen in the saturated and dried state. Here, following the strategy, 1D distributions of local porosity for the partly carbonated, leached, and sulfate-attacked specimens are all presented in Figure 7(a3–c3). The local porosity of the non-neutralized region was about 0.4 for all samples, but those of the carbonated, leached, and sulfate-attacked region changed to the range of 0.2 to 0.3, 0.7 to 0.8, and 0.4 to 0.5, respectively. The porosity result pointed out a refined microstructure by carbonation (so-called ‘pore clogging’ [49]), with a deteriorated one by leaching [47] or sulfate attack [54]. Specifically, focusing on the undulating porosity profile within the sulfate-attacked area, a layer-by-layer deterioration behavior during sulfate-attack was observed [12,54]. Even though the precision level of XRAM was lower compared to CT, as the sampling interval was 1 mm, the measured neutralization depth was still compatible with that based on indicator spray, and that, once again, confirmed the reliability of F. orchid indicator. In addition, a less increased porosity near the leaching front and a slight increase in porosity within the sulfate-attacked region were spotted, and both phenomena supported the existence of a porous leached/sulfate-attacked area, where pigment could be accumulated.

## 4. Discussion

### 4.1. F. Orchid Indicator in Characterization of Neutralization Front

Understanding the broadness of the neutralized (carbonated, leached, and sulfate-attacked) front is of high importance, as it can help unveil the kinetic mechanism behind each neutralizing progress [53,58]. For instance, if leaching was solely controlled by diffusion, loss of calcium-bearing materials would be faster compared with the invasion of water, and a narrow leaching front would, thus, be formed [46]. Meanwhile, if the time required for dissolution of calcium-bearing materials was slower, the leaching process was then not solely controlled by diffusion, and a broader leaching front was expected.

**Figure 8:**
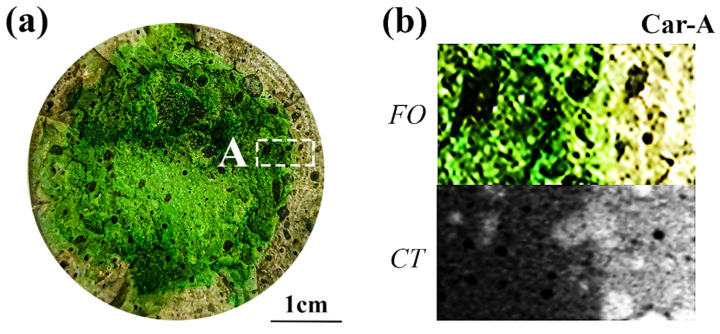

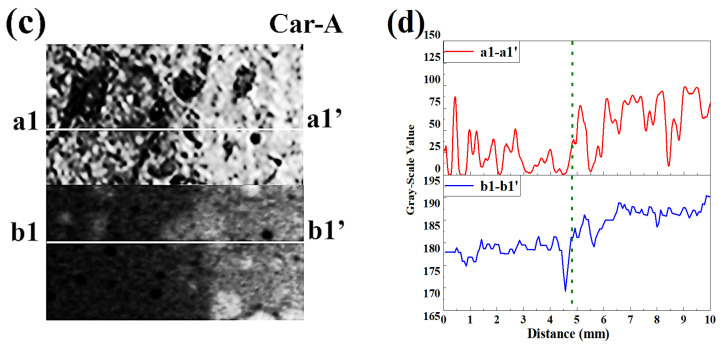
Partly carbonated specimen. (**a**) Cross-section sprayed with F. orchid indicator; (**b**) magnified local image of A and related CT image; (**c**) Binarized image of (**b**); (**d**) 1D distribution of grayscale value along a1-a1′ and b1-b1′ in (**c**).

Figure 8a shows the cross-section of the partly carbonated specimen after spraying with F. orchid indicator. To better demonstrate the details of the carbonation front, the local image and its related CT rendering were magnified, as shown in Figure 8b. Apparently, the carbonated region revealed though indicator spray showed high consistency with that revealed through CT, despite the boundary being relatively vague compared with that based on phenolphthalein. To quantitatively illustrate the scale of the carbonation front, the image in Figure 8b was binarized (see Figure 8c), and 1D distributions of the grayscale value were then plotted (see Figure 8d). As a sharp carbonation front was observable from the 1D grayscale value profile based on both the indicator spray and CT scan, the progress in the present study was revealed to be under the control of diffusion.

**Figure 9:**
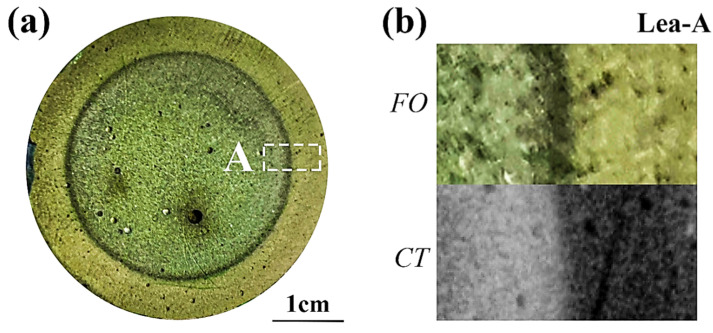

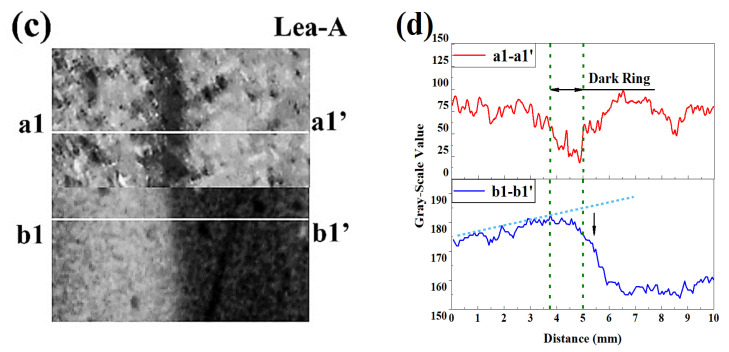
Partly leached specimen. (**a**) Cross-section sprayed with F. orchid indicator; (**b**) magnified local image of A and related CT image; (**c**) binarized image of (**b**); (**d**) 1D distribution of grayscale value along a1-a1′ and b1-b1′ in (**c**).

For leaching, following a similar strategy, the cross-section, magnified local image, binarized image, and 1D distribution of grayscale values are shown, respectively, in Figure 9a–d. Because of the dilution effect, the color difference between the leached and non-leached area was less significant. Fortunately, the leaching front was presented as a dark ring, connecting the non-leached and leached area. Even though calcium-bearing materials were partly lost within the leaching front (see the reduced grayscale value near the leaching front in Figure 9d, where a blue dashed guideline is presented showing the expected grayscale value without leaching), the pH of the leaching front was still insufficient to trigger discoloration of anthocyanin. Therefore, the leaching front remained green after spraying with F. orchid indicator. Regardless, the microstructure of the leaching front was more porous as compared to the non-leached area (see Figure 7(b3)), so a larger amount of anthocyanin could reside in this area, causing color accumulation (emergence of deep green). Read from Figure 9d, the width of the leaching front was estimated to be 1–2 mm.

**Figure 10:**
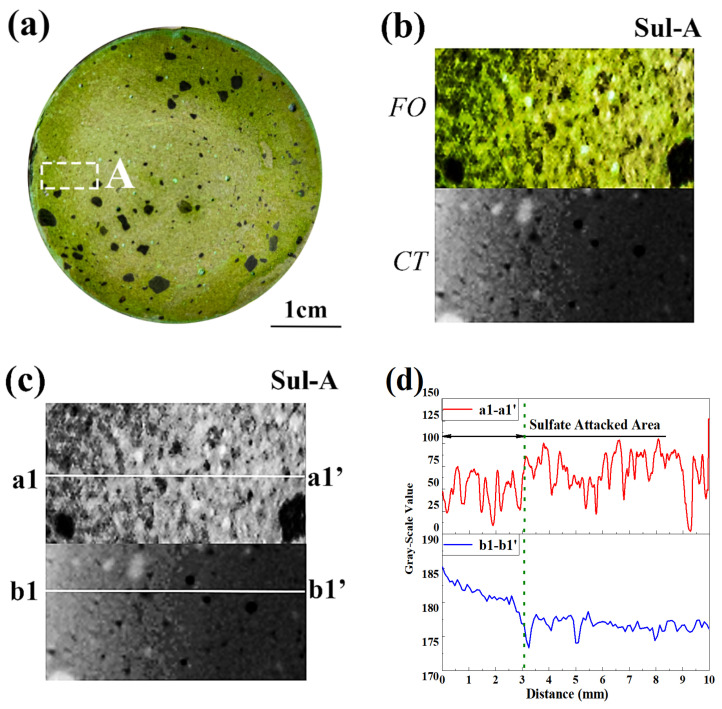
Partly sulfate-attacked specimen. (**a**) Cross-section sprayed with F. orchid indicator; (**b**) magnified local image of A and related CT image; (**c**) binarized image of (**b**); (**d**) 1D distribution of grayscale value along a1-a1′ and b1-b1′ in (**c**).

Figure 10 presents the cross section, local image, binarized image, and 1D distribution of grayscale values for the partly sulfate-attacked specimen. Similar to leaching, the efficiency of F. orchid indicator in characterizing the sulfate-attacked region was highlighted. Better yet, the obtained sulfate-attacked depth was compatible with that based on CT (see Figure 10d), which highlighted the effectiveness of F. orchid indicator in characterizing sulfate attack.

### 4.2. Applicability of F. Orchid Indicator in Neutralization Characterization

Although effective, the decision to replace phenolphthalein completely by F. orchid indicator in neutralization characterization should still be made very carefully. First, a more complex formulating procedure and a stricter preserving condition are adopted, so the cost performance of F. orchid indicator is lower than those of phenolphthalein, indigo carmine, malachite green, and thymolphthalein. Fortunately, F. orchid is a widespread ground cover plant in Asian countries. Therefore, its use as the indicator source would be cheaper compared to those sourced from fruit, vegetable, or flowers [38,39,40,41]. Secondly, the color intensity of phenolphthalein/thymolphthalein is higher compared to anthocyanin. Therefore, when observing irregular-shaped neutralization fronts (e.g., carbonation front), phenolphthalein/thymolphthalein, with higher color intensity, is a better choice.

**Figure 11:**
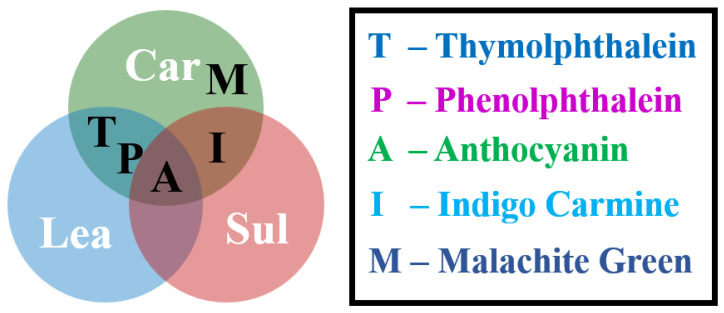
Suitable circumstances for each pH indicator.

The benefits of promoting F. orchid indicator are clear, because the F. orchid plant is nontoxic, and the indicator based on the F. orchid source is, thus, safer. In addition, due to weaker color intensity, color accumulation caused by pigment residing in porous media can be better observed by anthocyanin spray. Even though indigo carmine is also capable of catching the sulfate-attacked area, the indicator failed to clearly illustrate the leaching front. Based on the result, the newly developed F. orchid indicator is referred here as a universal pH indicator, which is effective in carbonation, leaching, and sulfate-attack characterization. Circumstances suitable for phenolphthalein, malachite green, indigo carmine, thymolphthalein, and newly developed F. orchid indicator (anthocyanin) are concluded accordingly in Figure 11.

## 5. Conclusions and Future Work

### 5.1. Conclusions

In this paper, anthocyanin extracted from F. orchid petals was used to characterize the neutralized (carbonated, leached, and sulfate-attacked) region of cement-based materials. To verify the reliability of the newly developed pH indicator, the neutralized region characterized by F. orchid indicator was examined by spraying with four widely used pH indicators (phenolphthalein, thymolphthalein, malachite green, and indigo carmine), CT, and XRAM. Combining current work on the durability, reliability, and applicable scope of F. orchid indicator, a ‘leap’ toward the application of anthocyanin in the durability design of concrete structures was made. Conclusions can be obtained here.

(1) Although the newly developed F. orchid indicator is less durable compared to phenolphthalein, the partly deteriorated indicator is still effective in neutralization characterization due to its stable discoloration pH.

(2) When observing the irregular-shaped neutralization front (e.g., carbonation front), F. orchid indicator is less preferable to phenolphthalein or thymolphthalein due to its low color intensity.

(3) When handling the partly leached specimen, the new F. orchid indicator is a better choice over phenolphthalein, thymolphthalein, indigo carmine, and malachite green, as it can uniquely plot the image of the leaching front.

(4) Compared with phenolphthalein, thymolphthalein, and malachite green, the new F. orchid indicator and indigo carmine are more suitable pH indicators in tracing the sulfate-attacked area through pigment accumulation.

(5) The carbonation front is narrow in the present study, suggesting that the progress is mainly controlled by diffusion, while for the leaching and sulfate attack, their broader fronts reveal that both progresses are co-controlled by diffusion and reaction.

### 5.2. Future Work

In the present study, F. orchid indicator was used as a pH indicator to trace the neutralization behavior of cement-based material. Although effective, the current work was limited to pastes made of pure cement, while for specimens cast from other types of cement, or blended with supplementary materials, the effectiveness of the new indicator remains unexamined. Therefore, further work on specimens cast from other types of cement, or blended with supplementary materials, should be carried out as well. In addition, current durability tests were carried out under accelerated conditions, and as the mechanism of the accelerated test is different from that in-site, specimens pretreated with natural carbonation, leaching, or sulfate attack should also be adopted to verify the reliability of F. orchid indicator. Furthermore, the cost performance of F. orchid indicator was relatively low at the current stage, so a better formulating/preserving strategy should be developed in the future to control the budget of indicator production. All work mentioned above continues in our laboratory.
